# Ten Years of Mediterranean Monk Seal Stranding Records in Greece under the Microscope: What Do the Data Suggest?

**DOI:** 10.3390/ani14091309

**Published:** 2024-04-26

**Authors:** Maria Solanou, Aliki Panou, Irida Maina, Stefanos Kavadas, Marianna Giannoulaki

**Affiliations:** 1 Hellenic Centre for Marine Research, Institute of Marine Biological Resources and Inland Waters, 71500 Heraklion, Greece; m.solanou@hcmr.gr; 2 Archipelagos—Environment and Development, Lourdata, 28100 Kefalonia, Greece; aliki.panou@yahoo.gr; 3 Hellenic Centre for Marine Research, Institute of Marine Biological Resources and Inland Waters, Anavyssos, 19013 Attica, Greece; imaina@hcmr.gr (I.M.); stefanos@hcmr.gr (S.K.)

**Keywords:** Mediterranean monk seal, stranding events, oscillation indices, fishing grounds, hotspot areas, breeding sites, eastern Ionian Sea

## Abstract

**Simple Summary:**

Ten years of Mediterranean monk seal stranding data along the Greek coastal areas were analyzed. The spatial distribution of stranding events along with the associated causes of death were modeled and mapped, while the relationship between the possibility of a stranding event and environmental and anthropogenic factors was thoroughly explored. The number of stranding events was found to be higher during the winter period. Moreover, the values of two major teleconnection oscillation indices during the first half of the year as well as the sea surface temperature in the Mediterranean from October to December were positively correlated with the number of stranding events of “unknown” cause. The size of fishing grounds and the number of sightings were associated with a higher probability of “human-related” stranding events. Finally, the central Aegean Sea was found to be a hotspot for “human-related” strandings, while, the eastern Ionian Sea, the southern coasts of the island of Cephalonia, and the gulf between the island of Lefkada and mainland Greece were found to present higher probabilities of monk seal subadult stranding events.

**Abstract:**

This paper presents the results of an analysis of stranding events of the Mediterranean monk seal *Monachus monachus* over a decade. The analysis involved categorization according to the cause of stranding and seasonality, the identification of hotspot stranding areas and an assessment of possible correlations between stranding events and environmental/climatic patterns using time series analysis. Moreover, Generalized Additive Models (GAMs) were applied to explore the effects of the size of small-scale fishing grounds, the number of species sightings, and the occurrence of reproduction sites on “human-related” strandings. Finally, special focus was put on the central part of the eastern Ionian Sea for the assessment of stranding hotspot areas by means of the Multi-Criteria Decision Analysis (MCDA) approach, based on different kinds of spatial information such as anthropogenic pressures and the location of breeding sites and feeding grounds. Time series analysis results revealed that oscillation indices, during the first half of the year, and sea surface temperature (SST) in the Mediterranean from October to December were positively correlated with monk seal stranding events. GAMs underlined that areas combining extended small-scale fishery grounds and a higher number of sightings were more likely to cause more strandings. Regarding spatial analyses, the central Aegean Sea was highlighted as a hotspot for “human-related strandings”, while the MCDA approach emphasized that the southern coasts of Cephalonia and the gulf between Lefkada and mainland Greece were susceptible to subadult strandings.

## 1. Introduction

The genus *Monachus* includes three geographically separated species: the Mediterranean monk seal *Monachus monachus*; the extinct Caribbean monk seal *Monachus tropicalis*; and the Hawaiian monk seal *Monachus schauinslandi*. The Mediterranean monk seal (*Monachus monachus*) is the only seal living in the Mediterranean Sea; with a global population of fewer than 1000 individuals, it was considered “Endangered” until recently, but its status was shifted to “Vulnerable” following the latest assessment by the International Union for the Conservation of Nature (IUCN) [1]. The species was once abundant throughout the Mediterranean and the Black Sea, but human persecution and habitat destruction have led to the shrinkage of the species’ historical range along with a severe decrease in the population [2]. Conservation efforts in Greek waters were initiated in the Northern Sporades in 1976 and were followed by a presidential decree in 1981 [3] and the establishment of a marine national park there in 1992. Another monitoring initiative started in the Ionian Sea in the mid-1980s [4,5]. Following a series of awareness-raising actions and monitoring projects during recent decades, the eastern Mediterranean monk seal subpopulation was recently estimated at around 444–600 mature individuals [1], forming actively reproducing groups distributed mainly along the mainland coasts and the islands of Greece and, to a lesser extent, Cyprus and the western and southern Turkish coasts ([2] and references therein). In Greece, the species is known to feed on a wide variety of fishes, cephalopods, and crustaceans (e.g., [6]), foraging in waters up to 200 m deep [7,8]. As pinnipeds, they rely on coastal haul-out areas during their annual life cycle. They used to inhabit open beaches, but currently use marine caves to give birth and raise their pups [2] due to disturbance and the continuous loss of available habitats, which is the result of increasing human activities—tourism in particular [4]. A recent encouraging sign of re-using open beaches by female monk seals and their pups was reported by Dendrinos et al. [9] and Panou (unpublished data).

One of the most important threats for the species in Greek waters is related to their interaction with fisheries (e.g., [2,4,5]). The interaction of monk seals with small-scale coastal fisheries often results in partial loss of fishers’ income due to damage to their fishing gear, and this also partly results in accidental entanglements ([2] and references therein). Damage caused by seals traditionally resulted in aggressive response by fishers often resulting in deliberate killings. Fishers consider that this may reduce the damage to their gear, as well as fish-catch losses due to depredation.

Pinnipeds, such as monk seals, are considered stranded when found dead on land or in the water or need medical attention. During the last decade, more than 230 stranded Mediterranean monk seals were recorded by port authorities on the Greek shores, either alive or dead. Stranding information can be one of the primary sources of evidence regarding the interaction between marine megafauna and human activities [10]. Such information can also provide a unique opportunity to improve our understanding of the causes of mortality, advance our knowledge of the population dynamics of the species (e.g., [11]), and define focus areas for protection measures and public awareness campaigns.

Here, we thoroughly explore the monk seal stranding events recorded in Greece over the last decade, focusing on the cause of death, derived from port authority reports, the associated photographic material, and the small number of available veterinary reports. All the cases where the cause of death could not be identified were assigned as “unknown”. For these cases, possible temporal trends related to environmental variability were explored based on time series analysis and major teleconnection indices, such as the North Atlantic Oscillation (NAO). The emphasis was placed on stranding events that were considered “human-related”, exploring the effect of small-scale fisheries along with other factors such as species sightings and the occurrence of reproduction sites. Furthermore, spatial analysis was applied to identify areas with high stranding occurrences, thus defining “hotspot” stranding areas.

Finally, additional analysis was carried out in the central part of the eastern Ionian Sea (hereafter the Ionian Sea), namely Cephalonia, Ithaca and the islets in the Inner Ionian Archipelago. The area was selected as it is one of the best-studied areas in Greece where a monk seal sub-population lives and breeds, consisting of a minimum of 25 adult/subadult photo-identified monk seals (Aliki Panou, unpublished data), several juveniles and up to 12 pups born annually [12]. Here, conservation efforts date back to the mid-80s [13], including monitoring the interaction between monk seals and the small-scale fishery, recording the damage to catches and gear and the cause of seal deaths for the first time [4]. The local population of this area may serve as a nucleus from which the entire Adriatic–Ionian Basin could be re-colonized [12] given the ability of the Mediterranean monk seal to travel up to 300 km within a few months [8]. The Multi-Criteria Decision Analysis (MCDA) approach was applied [14] here as an indirect way to identify potential regions with high occurrence of monk seal stranding events based on different types of spatial information. The approach has been gaining recognition during the last decade and is usually coupled with GIS tools to enhance the application of spatial decision-making and management planning in various environmental fields (e.g., [15,16,17]). Here, sources of anthropogenic pressure such as small-scale fishing, possibly favoring the possibility of a stranding event, were combined with factors that may benefit monk seal populations and, thereby, hinder the number of strandings of “unknown” cause of death. The results are discussed within the framework of conservation plans and management measures to control anthropogenic threats to the species, such as entanglement and other sources of human-caused mortality or stress. 

## 2. Materials and Methods

### 2.1. Data Mining: Cause of Death

**Figure 1:**
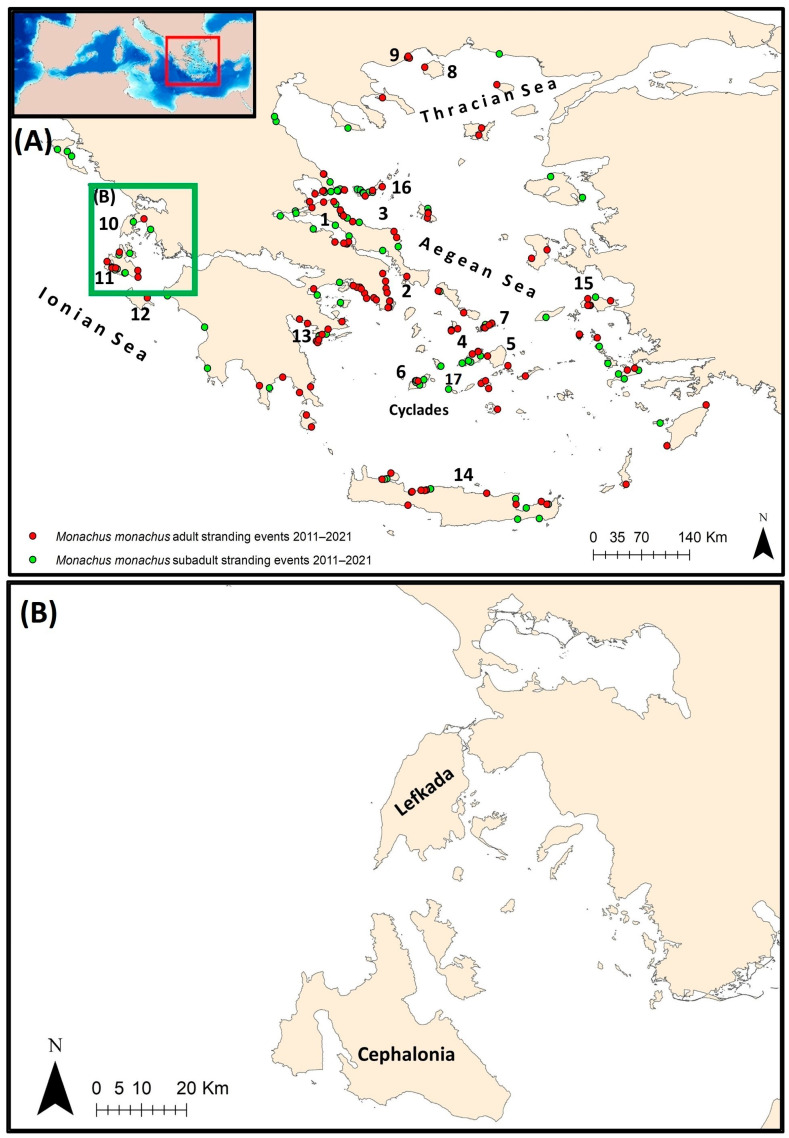
(**A**) Map of the wider study area in Greece and Mediterranean monk seal stranding events. (**B**) Map of the central part of the Ionian Sea where MCDA was applied. Toponyms mentioned in the text are shown: 1 North Evoikos Gulf; 2 South Evoikos Gulf; 3 Eastern Evia; 4 Paros; 5 Naxos; 6 Milos; 7 Mykonos; 8 Thasos; 9 Kavala; 10 Lefkada; 11 Cephalonia; 12 Zakynthos; 13 Argolikos Gulf; 14 Crete; 15 Samos; 16 Sporades; 17 Folegandros.

Data on marine mammal strandings derived from port authority reports have been submitted to the National Network for Monitoring Stranding Events during the period 2011–2021 and have been stored in a dedicated database of the Institute of Marine Biological Resources and Inland Waters (IMBRIW) of the Hellenic Centre for Marine Research (HCMR). Data included more than 230 stranding events of clearly identified animals reported as Mediterranean monk seals (Figure 1A). The island of Zakynthos was not included in the analysis due to reporting issues. All stranding records were georeferenced based on toponym information and an ArcGIS, version 10.4 [18], point shapefile was produced.

Stranding events were initially categorized into “Dead” or “Alive”. Subsequently, they were categorized, based on the cause of death, into “unknown” (i.e., no information available about the cause of death according to the veterinary report, port authority reports and/or evidence based on photographs) or “human-related”. As veterinary reports were available in less than 5% of the cases, and necropsies were even rarer, the cause of death was considered to be “human-related” only in the cases where an obvious human intervention, capable of causing direct death, had occurred (i.e., one or more severe wounds or suffocating fishing gear leftovers were apparent based on photographs or clearly stated in the port authority report). Thus, the “human-related” stranding events were further classified into the following categories: “fishing gear entanglement”, “wounded”, “gunshot” and “internal bleeding (due to dynamite)”. 

Moreover, the quality of information about sex and size varied depending on different factors such as proximity to the stranding point, degree of corpse decay, experience of the port authority personnel and quality of photographic material. All these often resulted in a lack of information or doubtful output for numerous cases, especially regarding sex determination. Consequently, analysis per sex was not applied. Moreover, it was considered more reliable to roughly assign records to “subadults” or “adults”. Finally, seasonal stratification was applied and stranding events were classified as “winter/ cold season” (i.e., October to April) or “summer/ warm season” (i.e., May to September) incidents. 

### 2.2. Temporal Trends in Stranding Events Related to Environmental Variability

Minimum/maximum Auto-correlation Factor Analysis (MAFA) [19] was applied to identify any correlation between stranding events of “unknown” cause of death (dependent variable) and environmental variables (independent variables). Here, MAFA was applied to test the correlation between monk seal strandings and the time series from 2011 to 2021 of (i) the sea surface temperature (SST, https://oceancolor.gsfc.nasa.gov/ accessed on 20 January 2024) as 3-month running mean at Mediterranean Basin level as well as at Greek Seas level since 2011, (ii) the El Nino Southern Oscillation estimated as the trimonthly mean values of the index (ENSO, https://psl.noaa.gov/enso/mei/ accessed on 20 January 2024), (iii) the North Atlantic Oscillation estimated as the bimonthly mean values of the index based on the difference in normalized sea level pressures between Ponta Delgada, Azores, and Stykkisholmur/Reykjavík, Iceland (NAO, https://www.ncdc.noaa.gov/teleconnections/nao/ accessed on 20 January 2024), and (iv) the sea surface chlorophyll (Chla, https://oceancolor.gsfc.nasa.gov/ accessed on 20 January 2024) as annual values and seasonal values over the continental shelf of the Mediterranean, including the Greek Seas. NAO was estimated as bi-monthly values, as this allows visualization of the index impact throughout the year and is based on the surface sea-level pressure difference between the Subtropical (Azores) High and the Subpolar Low in the Atlantic Ocean. NAO exhibits considerable intersessional and interannual variability, and prolonged periods (several months) of both positive and negative phases of the pattern are common. Strong positive phases of NAO tend to be associated with below-normal temperatures often across southern Europe and the Middle East. Opposite patterns of temperature and precipitation anomalies are typically observed during strong negative phases of NAO. ENSO reflects a naturally occurring anomalous state of tropical Pacific coupled ocean-atmosphere conditions. It is the primary predictor of global climate disruptions. These can persist over several seasons and thereby produce severe regional effects. The Oceanic Niño Index (ONI) is given as a 3-month running mean of the Extended Reconstructed SST anomalies (ERSST.v5) derived from NOAA National Climatic Data Center regarding the Niño 3.4 region (5oN-5oS, 120o-170oW). 

MAFA is a type of principal component analysis (PCA) for short time series for extracting trends and estimating index functions from multiple time series as well as a signal extraction procedure [20]. It provides a method for analysis of multivariate time series, in which the information on the time ordering of the change is used [21]. The MAFA technique selects linear combinations of the original variables that have the largest lag-one autocorrelation. The significant MAFAs provide the smoothest representation of the contrasts in variables over time and each MAFA is uncorrelated, such that they each represent a different mode of variation in multivariate time series [19,21,22]. The axes represent a measure of autocorrelation. While in PCA, axes explain variance, with PCA-axis 1 typically explaining most variance, the first MAFA axis has the highest autocorrelation, and because trends are associated with high autocorrelation, it represents the main trend of data [20,23]. As in PCA, loadings are estimated and used to infer which response variable is related to a particular MAFA axis, by cross-correlation between MAFA axes and each of the original explanatory time series. The possible effect of other time series, (i.e., explanatory variables) can be examined through cross-correlation between MAFA axes and explanatory variables [20]. The loadings determine the relationship between individual response variables and particular MAFA axes. We used the Brodgar v.2.7.5 (http://www.brodgar.com) software package to carry out MAFA of the time series of response variables and explanatory variables, and significance was assessed at *p* < 0.05 in all cases.

### 2.3. Spatial Analysis: Identifying Hotspot Stranding Areas

The Kernel Density analysis tool in ArcGIS was applied to provide a visual representation of hotspot stranding areas in Greek waters regarding: (a) stranding events of “unknown” causes of death and (b) “human-related” ones. The Kernel Density tool calculates the density of features in a neighborhood around those features. It was calculated for point features as the density of point features around each output raster cell. Conceptually, a smoothly curved surface is fitted over each point. The surface value is highest at the location of the point and diminishes with increasing distance from the point, reaching zero at the “search radius distance” from that point. Here, the search radius distance was defined at 10 nautical miles (nm) in the case of total strandings and at 20 nm in the case of “human-related” strandings, considering the complex topography of the Greek Seas along with the number of available records. The Kernel function is based on the quartic kernel function described in Silverman [24]. The search radius is based on Silverman’s Rule-of-thumb bandwidth estimation formula but it has been adapted for two dimensions. This approach of calculating a default radius generally avoids the ring around the points, a phenomenon that often occurs with sparse datasets and is resistant to spatial outliers, i.e., a few points that are far away from the rest of the points.

### 2.4. Identifying Factors Related to the Spatial Pattern of Monk Seal Strandings

**Figure 2:**
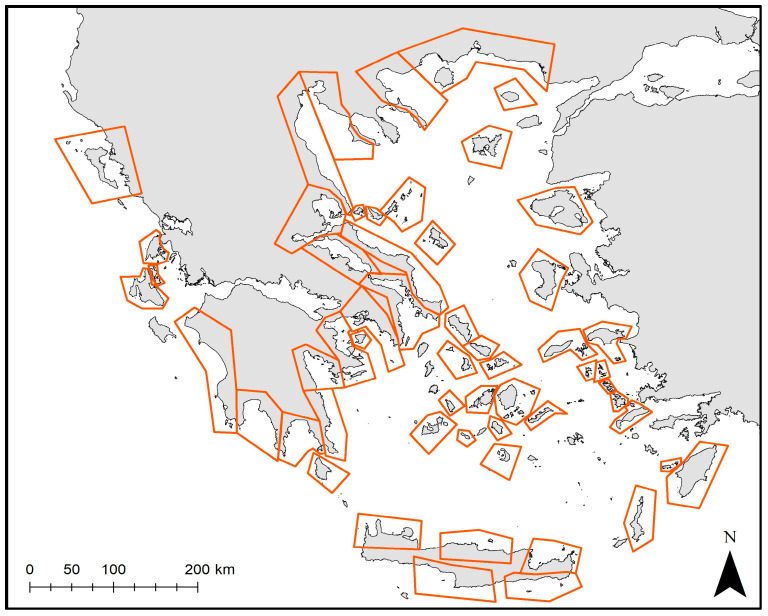
Division of the Greek coastline into subareas for analysis purposes.

To assess the effect of different factors (such as the size of fishing and feeding grounds or the number of sightings) on stranding numbers, Generalized Additive Models (GAMs) were applied. For this purpose, the Greek coastline was divided into subareas, considering the complex topography of the region and the numerous islands (Figure 2) and the average annual strandings per subarea were calculated along with the following factors:

- The size of fishing grounds of the small-scale fishery (SSF): the fishing area that corresponds to the upper 50% of the accounted small-scale fishing density estimated as the average for the 2011–2021 period (based on [17]).
- The size of potential feeding grounds: the estimation was based on the catch of undersized discarded fish from commercial bottom trawls (modeled based [25,26]). Based on these data, the feeding area that corresponds to the upper 25% of the accounted coastal bony fish biomass was estimated and assigned per subarea.
- Monk seal sightings: Sightings might be considered as a rough proxy of the distribution or as an indicator of the activity of the monk seal population in the Greek Seas. Based on Dendrinos et al. [27], a tentative number of sightings was assigned per subarea, ranging from 10 to 150 sightings.
- Breeding sites (including potential and confirmed ones) and resting sites. The level of density of existing caves (based on [27]) was assigned per subarea. We acknowledge the fact that available information can be less accurate for certain areas; however, it is the best information currently available.

Subsequently, GAMs [28] were applied, using stranding numbers as the dependent variable whereas the size of fishing and feeding areas, the frequency of monk seal sightings and marine cave density were used as explanatory variables. The analysis and the selection of the GAMs smoothing predictors were carried out using the MGCV library [29] in the R statistical software, version 4.0.3 [30]. In each model fit, a double penalty was applied to the penalized regression solved by MGCV, which allows variables to be solved out of the model entirely [31], being more robust in identifying important features. The degree of smoothing was chosen based on the observed data and the restricted maximum likelihood (REML) estimation as suggested by Marra and Wood [31]. The best model was selected based on the minimization of Akaike’s information criterion (AIC) and the level of deviance explained (0-100%; the higher the percentage, the more deviance explained). To avoid over-fitting and to simplify the interpretation of the results, the REML method was applied, and the maximum degrees of freedom (measured as the number of knots k) allowed for the smoothing functions were limited to the main effects at k = 5. In the case of the overall number of strandings, the Poisson error distribution with the log link function was used and the natural cubic spline smoother [28] was applied for smoothing the independent variables and GAM fitting. In the case of the number of “human-related” strandings, the zero-inflated Poisson error distribution with the identity link function was used. Validation graphs (e.g., residuals versus fitted values, QQ-plots and residuals versus the original explanatory variables) were plotted to detect the existence of any pattern and possible model misspecification. Residuals were also checked for autocorrelation. The output of the final selected GAM is presented as plots of the best-fitting smooths. 

### 2.5. Identifying Areas with High Susceptibility to Subadult Strandings at Small-Scale Basis: Focusing on the Central Part of the Ionian Sea

Multi-Criteria Decision Analysis (MCDA) [14,32] was applied in the central part of the Ionian Sea (Figure 1B), using independent information, to identify coastal areas susceptible to monk seal strandings. The output was evaluated in terms of the existing local information on strandings. The synergetic capabilities of GIS and MCDA can be considered as a process that transforms and combines geographical data and value judgments to obtain information for decision-making, providing benefits for advancing theoretical and applied research [33]. The approach involves the simultaneous use of multiple and sometimes conflicting criteria and information sources (both qualitative and quantitative). The analytical hierarchy process (AHP), proposed by Saaty [34], constitutes a widely used MCDA method that was selected within the framework of the current study. AHP compares the importance of all evaluation criteria by creating a set of pair-wise comparisons on a scale of 1 to 9; 1 denotes those two factors as equally important, while 9 considers that one factor is extremely more important than the other. These comparisons are used to obtain the weights of importance for the decision criteria [34,35]. Specifically, the process is divided into four steps: (i) constructing the hierarchy of the indicator system, (ii) creating a pairwise comparison matrix; (iii) calculating the ranking of the level and obtaining the maximum eigenvalue λ_max_ and (iv) calculating the consistency ratio (CR) [36]. When CR < 0.1, the consistency of the results is considered acceptable; otherwise, pairwise comparisons should be applied again, changing the relative importance of the factors.

Here, AHP was applied to generate a priority ranking of (i) factors of pressure, favoring the possibility of a stranding event, and consequently assigned a positive (+) correlation, and (ii) factors benefiting the good population status of monk seals and, thereby considered to hinder strandings and assigned a negative (−) correlation (Table 1). Factors favoring the stranding events comprised (a) small-scale fishing grounds (based on [17]) and (b) the distance from NATURA 2000 sites (https://emodnet.ec.europa.eu/en/map-week-marine-natura-2000-sites accessed on 20 October 2023), considered as important marine protected areas. The greater the distance from a NATURA 2000 site, the greater the possibility of anthropogenic activities that may induce stranding events. Therefore, this factor was positively correlated with the results. As surveillance remains minimal at most marine NATURA 2000 sites in Greece, the factor was given a very small contribution percentage in comparison to other factors. Similarly, fishing grounds were thought to possibly favor stranding events, since they are likely related with a higher possibility of interaction of the species with fishers. On the contrary, factors favoring good population status included (a) distance from marine caves that are known to constitute highly disturbed breeding sites for the species (information provided by Aliki Panou), (b) distance from aquaculture infrastructures (https://emodnet.ec.europa.eu/en/human-activities accessed on 20 October 2023) and (c) feeding grounds (based on the modeled catch of undersized discarded fish from commercial bottom trawls, [25,26]). Specifically, breeding caves that were highly disturbed by anthropogenic activities during recent years were considered to favor stranding events. This is reflected in the high percentage (46%) of stranded subadults (body length < 170 cm). Ranking of existing marine caves was based on expert knowledge (Panou, personal communication). Aquaculture premises may attract monk seals, often triggering the aggressive response of the owners. Since the importance of aquaculture for monk seal deaths and consequent strandings remains unknown, this factor was also attributed to a small contribution percentage in comparison to the others. Since proximity to the above specific marine caves and aquaculture premises implies more stranding events, both factors were assigned a negative (−) sign. Regarding the feeding grounds, they favor good population status by providing the species with food. Thus, they were considered to negatively impact stranding events and assigned a negative (−) sign.

The five factors that were selected were further resampled and analyzed with a spatial resolution of ~1.313 × 1.313 km under a gridded ArcMap 10.4 environment. Each one of them was further assigned a grading value which was subsequently reclassified in a common 1 to 5 scale of importance (based on the literature and expert judgement). In the next step, AHP was applied, and relative weights were calculated for each factor (Table 1). Finally, a map highlighting areas prone to monk seal strandings, especially subadults, was produced through an overlap of the aforementioned layers.

## 3. Results

### 3.1. Data Mining: Cause of Death

A total of 236 Mediterranean monk seal stranding events were recorded during the period 2011 to 2021. Based on the measurements recorded in port authority reports, up to 54% of stranded animals were considered adults (body length > 170 cm) and 46% as subadults (body length < 170 cm), in accordance with Karamanlidis et al. [2]. Most records involved dead animals (222 dead vs. 14 alive animals), while four cases of alive stranding events (28%) were recorded as weak or malnourished. Regarding the dead animals, 81% of deaths were considered as of “unknown” cause, whereas 19% were considered “human-related”, associated with some sort of human interaction. Viral infection and the associated poor physical condition of animals are reported as additional causes of stranding or death, although representing less than 1% of the cases. The majority of “human-related” strandings were recorded as wounded (with one or multiple apparent serious wounds caused by a sharp object), followed by entangled (apparent signs of fishing gear, i.e., nets or fish hooks) and, finally, gunshot animals. These are summarized in Table 2, as identified based on either port authority or veterinary reports or on visual inspection of available photographic material. 

**Figure 3:**
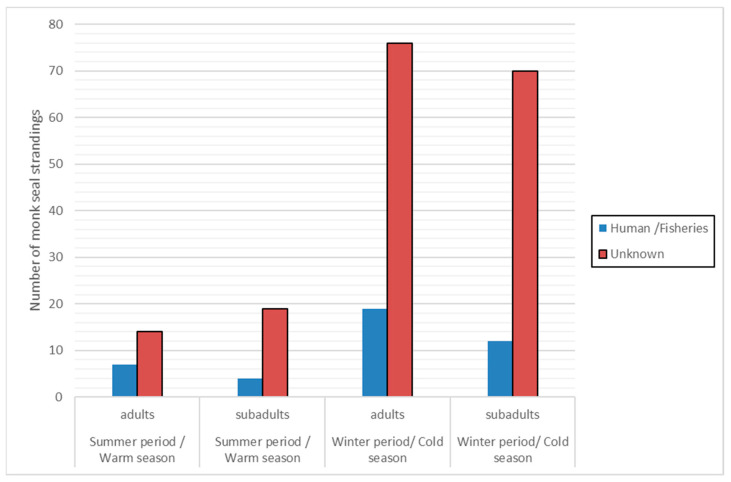
Number of monk seal strandings (adults and subadults) as reported during the period 2011–2021 in Greece. “Winter period/Cold season”: October–April, “Summer period/Warm season”: May–September.

The seasonal distribution of monk seal stranding events (adults and subadults) was divided into “winter/cold season” (i.e., months October to April, as the period dominated by rough sea conditions) and “summer/warm season” (i.e., May to September) (Figure 3). Results indicate a more than four times higher number of strandings due to “unknown” cause of death during the “winter/cold season”, for both adult and subadult individuals.

Stranding events due to “unknown” cause of death were more frequent in the “winter/cold season” while the difference between the two periods regarding the “human-related” strandings was much smaller. It is also apparent that the percentage of subadult strandings was much higher during the “winter/cold season”.

### 3.2. Temporal Trends in Stranding Events Related to Environmental Variability

**Figure 4:**
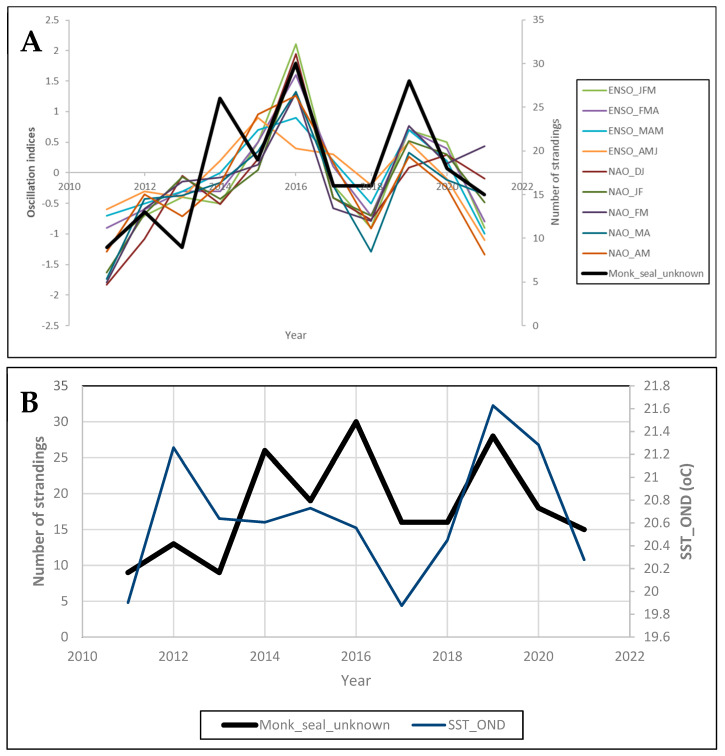
MAFA output with the significant correlations between monk seal “unknown” strandings and (**A**) Oscillation indices (ENSO_JFM: mean ENSO January to March, ENSO_FMA: mean ENSO February to April, ENSO_MAM: mean ENSO March to May, ENSO_AMJ: mean ENSO April to June, NAO_DJ: mean NAO December to January, NAO_JM: mean NAO January to March, NAO_MA: mean NAO March to April, NAO_MA: mean NAO April to May) and (**B**) SST (SST_OND: mean SST from October to December over the Mediterranean).

The MAFA output showed that autocorrelations were not significant for either the MAFA1 or the MAFA2 axis (*p* > 0.377) with no apparent trend in the scores of all axes (Figure 4). However, significant correlations were estimated between the monk seal strandings characterized by “unknown” cause of death and the tri-monthly ENSO estimates from January to June, the bimonthly NAO estimates from December to May (Figure 4A) as well as the trimonthly SST from October to December over the Greek Seas (Figure 4B). Thus, we observed that the oscillation indices during the first half of the year were positively correlated with monk seal strandings (i.e., more strandings with increased NAO and ENSO values), while the SST in the Mediterranean during the last part of the year shows a significant positive correlation, implying more strandings upon higher SST values from October to December. 

### 3.3. Spatial Analysis: Identifying Hotspot Stranding Areas

**Figure 5:**
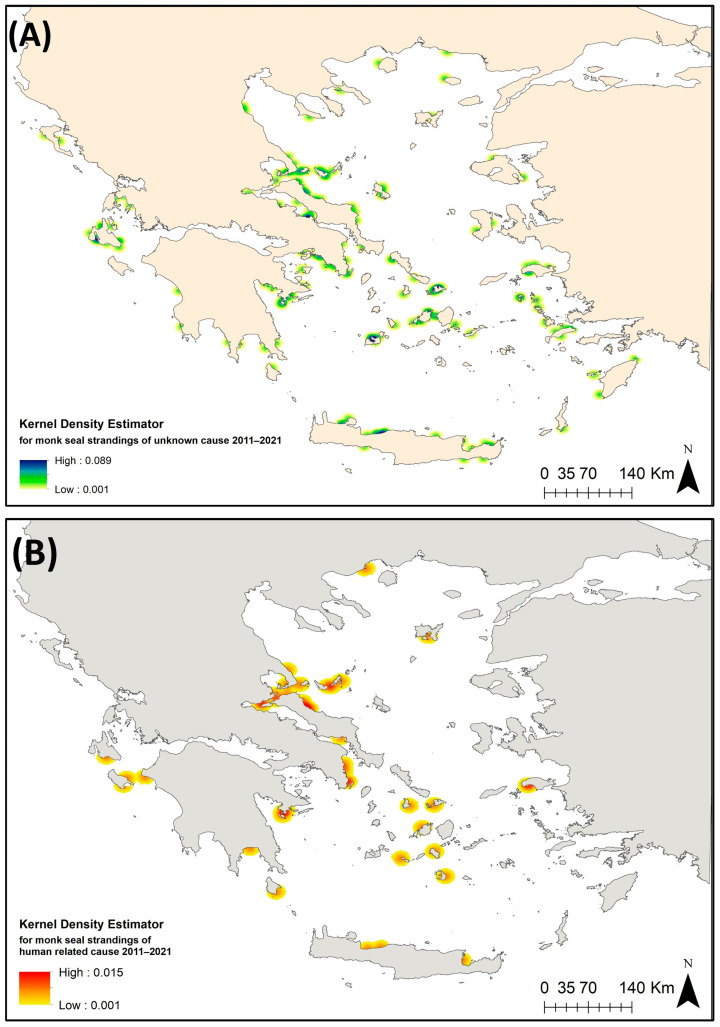
Kernel density-derived maps during the period 2011–2021 regarding: (**A**) Monk seal stranding events of “unknown” cause of death (both adults and subadults). (**B**) “Human-related” stranding events (both adults and subadults).

The toponym associated with each stranding record was turned into georeferenced information and Kernel density maps of the stranding events with “unknown” as well as those due to “human-related” cause of death were created in ArcGIS (Figure 5). These density maps verified as hotspot stranding areas for the “unknown” events in the area around Cephalonia, Ionian Sea. In the Aegean Sea, hotspots were shown in the northern part of the North Evoikos Gulf, the islands of Mykonos and Milos as well as the area between Paros and Naxos in the Cyclades and the coast of the northcentral part of Crete (Figure 5A). Hotspots of the “human-related” stranding events were identified in the wider area of North Evoikos Gulf–Sporades archipelago—the eastern coast of Evia, the area between the town of Kavala and Thasos in the Thracian Sea, the western part of South Evoikos Gulf, the outer part of Argolikos Gulf, the island of Samos in the eastern Aegean Sea and the area between Zakynthos and the mainland, Ionian Sea (Figure 5B).

### 3.4. Identifying Factors Related to the Spatial Pattern of Monk Seal Strandings

**Figure 6:**
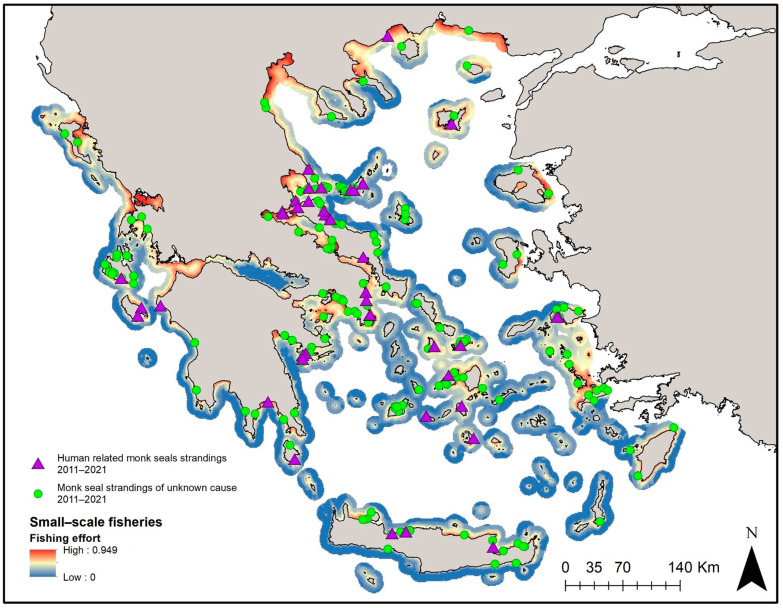
Small-scale fisheries’ fishing efforts and monk seal stranding events grouped into “human-related” and “unknown” cause of death.

**Figure 7:**
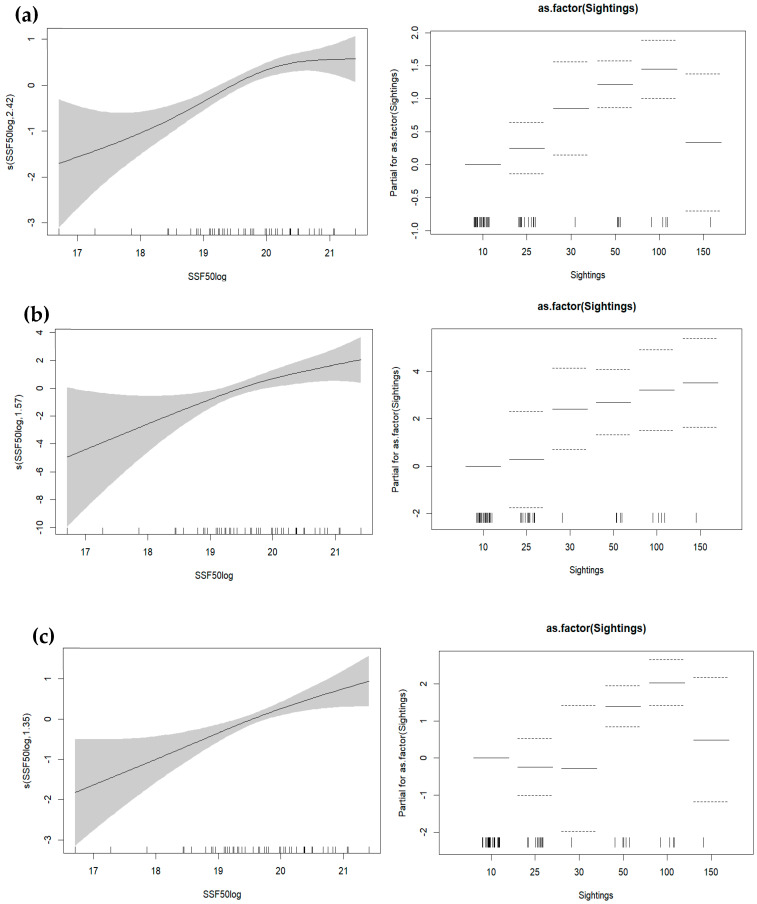
GAM plot showing the effect of the size of small-scale fishery fishing grounds (logSSF50) and monk seal sightings (**a**) total strandings (including adults and subadults), (**b**) “human-related” strandings (including adults and subadults) and (**c**) subadults strandings. Dashed lines denote confidence intervals.

Prior to GAMs, the exploratory analysis applied to identify possible correlations between the independent variables revealed a high degree of correlation between the size of the potential feeding grounds and the size of SSF grounds. Therefore, the size of SSF grounds (Figure 6) was kept for the analysis given that it is considered to be associated with human impact. GAM results indicated that a higher number of total stranding events was significantly associated with more extended fishing grounds combined with “50 to 100” sightings (Figure 7a, Dev. Explained 67.7%, *p* < 0.001, AIC = 211.150). This was also the case for the subadult stranding events (Figure 7c, Dev. Explained 56.9%, *p* < 0.001, AIC = 163.89). In the case of “human-related” stranding events, GAMs showed that a higher number of stranded records was associated with more extended fishing grounds combined with an increasing number of sightings (Figure 7b, Dev. Explained 58%, *p* < 0.001, AIC = 103.53). It is evident that areas with more extended fishing grounds for the SSF and more sightings are more likely to present higher numbers of monk seal strandings.

### 3.5. Identifying Important Areas at Small-Scale Basis: Focusing on the Ionian Sea

**Figure 8:**
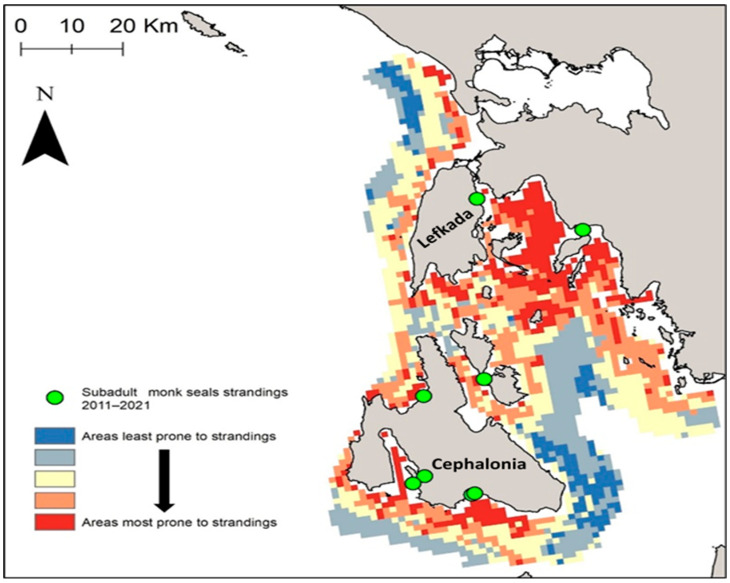
MCDA results combined with the positions of subadult monk seal strandings in the central Ionian Sea during the 2011–2021 period.

The relative importance of each criterion (based on expert knowledge and literature review) and the importance of the weights for each criterion (based on the pair-wise comparison matrix produced by the AHP method) are presented in Table 3. The consistency of the results is considered acceptable as CR = 0.03 < 0.1. Based on the overlap and weighted summation of all criteria datasets, a suitability map was produced (Figure 8). As subadult monk seals are more likely to relate to factors such as breeding and feeding sites, the positions of subadult monk seal strandings are also shown in Figure 8 in order to underline hotspot areas more susceptible to subadult monk seal strandings. Such areas include mainly the southern coasts of Cephalonia and Lefkada and the innermost gulf between Lefkada and mainland Greece (Figure 8). 

## 4. Discussion

The first step required for the conservation of the Mediterranean monk seal is to set effective management measures for controlling anthropogenic threats. An analysis of stranding events can contribute significantly to the determination of “human-related” sources of mortality such as entanglements or wounds. In general, fisheries constitute an important issue, as they compete with the Mediterranean monk seal for the same resources, which entails a hostile attitude of some fishers towards the species. Fishers have been killing monk seals throughout the Mediterranean Basin because of the considerable damage that the species may cause to catch and gear [4,5]. In Greece, accidental entanglements in fishing gear along with deliberate killings have been and still are the main causes of human-induced mortality [4,37]. 

Despite the fact that the very low percentage of available postmortem necropsies is a limitation of the study, an analysis of monk seal stranding events can shed light on the importance of species interaction with small-scale fisheries. Ten years of stranding analysis (2011 to 2021) in the Greek Seas showed that 19% of 236 stranding records were considered as “human-related”, associated with some sort of human interaction. The majority of “human-related” stranding events refer to “wounded animals” (up to ~47%) presenting one or more serious wounds, followed by “fishing gear entanglement” (~25%) and “gunshots” (~21%). Previous findings had highlighted a strong correlation between sightings and damage to fishing gear, i.e., seals roaming around (reported by observers) are likely to interact with small-scale fisheries [4], thus also implying a potential higher number of strandings. GAM analysis underlined, for the first time, that areas with more extended SSF fishing grounds and more sightings are more likely to present a higher number of “human-related” stranded animals. Density maps of “human-related” stranding events underlined higher stranding densities in the wider area of North Evoikos Gulf–Sporades Islands—the eastern coast of Evia, the area between the town of Kavala and Thasos in the Thracian Sea, the western part of South Evoikos Gulf, the outer part of Argolikos Gulf, the island of Samos in the eastern Aegean Sea and the area between Zakynthos and the mainland in the Ionian Sea. At such significant stranding areas, special efforts should be made to implement management measures and conduct public awareness campaigns. 

It should be highlighted that the actual number of “human-related” stranding events is most probably higher, but this cannot be ascertained due to the lack of veterinary reports for a large number of incidents, while there are specific types of anthropogenic impacts on marine life, possibly leading to strandings, which cannot be revealed without further investigation. For example, plastic ingestion has been identified as a major threat to marine mammals either by damage to the digestive system or by the release of toxic components [38,39]. The results of a recent study conducted using monk seal feces samples taken from caves in Zakynthos revealed microplastics and plastic additives in all samples investigated [40]. 

Mapping the probabilities of stranding events based on habitat suitability modeling, over surveyed and non-surveyed areas can be promising as a tool to determine important interaction areas for the species. Within this framework, the applied GAMs indicated that the numbers of total strandings were higher in the case of more extended fishing grounds and above a certain level of sightings (i.e., “50 to 100” of sightings). This was also the case for subadult strandings. Kernel density output maps of strandings of “unknown” cause further underlined the importance of the area around Cephalonia in the Ionian Sea. In the Aegean Sea, hotspots were shown in the northern part of North Evoikos Gulf, the islands of Mykonos and Milos, and also the area between Paros and Naxos over the Cyclades plateau and the coast of the northcentral part of Crete (Figure 5A). Subadult stranding events seem to be particularly abundant in the northern part of Evia, the northern part of North Evoikos Gulf, and around the area of Milos, Kimolos, Folegandros and Mykonos over the Cyclades plateau (Figure 1A). The images highlight the need to implement actions to reduce interaction with small-scale fisheries over the wider area of the Cyclades plateau and the northern part of North Evoikos Gulf–Sporades Islands as well as the eastern coast of the Evia. Such actions may include the avoidance of fishing methods likely to cause harm, the adoption of fishing gears that are likely to reduce bycatch as well as compensation measures for catch and gear damage that may diminish the hostile attitude of fishers

The results also show that the percentage of strandings, irrespective of cause of death, was found to be higher in the “winter/cold season” considering that the combination of rough sea conditions along with wounds caused by entanglement could result in a higher probability of drowning. This applies especially in the case of subadults as the percentage of subadult strandings was around three times higher in the “winter/cold season” compared to the “summer/warm season”. 

It is evident that a high percentage (81%) of monk seal stranding events remains “unknown” with respect to the cause of death. This is either because no necropsy was performed, or the causes of death are not easy to identify. Since extensive predation has not been documented as a major cause of mortality for the Mediterranean monk seal, only a low percentage of mortality is likely to be assigned to natural causes. Going forward, exploring monk seal movement patterns and foraging behavior could provide a broader context for understanding stranding patterns.

For the first time, we explored the temporal trend of Mediterranean monk seal strandings of “unknown” cause of death with respect to oscillation indices and temperature patterns. The latter may function as proxies of food availability (changes in climate and oceanographic conditions may affect pinnipeds by changing prey availability), sea level rise and the vulnerability of monk seals to diseases, factors that can affect monk seal mortality. For example, the effects of the El Niño Southern Oscillation events on the fur seal, sea lion, and harbor seal populations along the west coast of South and North America are well known [41]. Moreover, sea level rise may also pose a future threat to the survival of the Mediterranean monk seal as critical caves can be left with no access through the water [12]. Recent studies also highlight the possible long-term effect of climatic fluctuations on the demographic history of the species (e.g., the lowering of sea level and sea surface temperatures along with salinity variations that the Last Glacial Maximum provoked in the Mediterranean Basin ([42] and references therein), yet their role is still poorly understood. Our analysis revealed significant correlations (Figure 4) between the strandings of “unknown” cause of death and large-scale climatic patterns (i.e., tri-month ENSO estimates from January to June and the bimonthly NAO estimates from December to May) over the Greek Seas, implying a higher number of monk seal strandings with the higher phases of the NAO and ENSO indices during the first half of the year. Higher phases of NAO and ENSO are related to colder temperatures and cloudiness over the Greek peninsula and the surrounding waters [43]. Also, this period coincides with rough sea conditions, often associated with a higher probability of subadult drowning. Antonelis et al. [44] found that in the Hawaiian monk seal, pup girth at weaning was greater in El Niño years suggesting that short-term fluctuations in ocean conditions influence the species. This could be related to fluctuations in fish populations [45] and possibly reduced food availability during winter–spring, the energy-demanding reproduction period of the species. Regarding SST, a significant positive correlation during the last part of the year in the Mediterranean was calculated, implying the occurrence of a higher number of strandings upon higher SST values from October to December. Despite the general pattern of higher strandings during the winter period when rough sea conditions are likely to occur, MAFA has shown that within the available time series, this is more pronounced when higher temperatures occur. Higher temperatures are known to foster the development of pathogens [46] such as the morbili virus, thereby increasing the vulnerability of animals to infections. Warming temperatures can also affect the composition of prey communities and subsequently species diet or cause the deterioration of foraging habitats such as seagrass meadows [47]. Food limitation is known to result in low survival in the case of juvenile Hawaiian monk seals [48], possibly attributed to variable oceanographic productivity and interspecific competition [44,49,50]. Measures to re-direct the fishing sector towards sustainable practices should be considered in collaboration with the responsible fisheries authorities. Moreover, although it is not clear to what extent food shortage may significantly have affected the Mediterranean monk seal, the establishment of marine protected areas and/or no-take zones may partly counterbalance the reduction in marine resources.

With respect to the above, special focus was placed on the subarea of the islands of Cephalonia and Lefkada in the central Ionian Sea. MCDA highlighted areas over the southern coasts of Cephalonia and Lefkada and the innermost gulf between Lefkada and mainland Greece, which are susceptible to subadult strandings. Measures should be implemented to reduce threats associated with mortality or disturbance of mother–pup pairs and the disturbance of hauled-out seals in ways that could adversely affect their survival, behavior, birth rate or population growth. Most importantly, there is a general need for policies to avoid fishing methods most likely to cause harm along with possible fishing restrictions close to breeding caves. Long-term public awareness actions to increase public knowledge and citizen science to increase community involvement may mitigate the levels of friction. However, as stated by Panou et al. [12], the problem will not be solved unless beneficial measures for fishers are implemented while marine resources remain at low levels, especially if monk seal numbers are to increase due to effective protection measures.

Although stranding events constitute a cost-effective source of information, they are associated with high uncertainty in terms of the recorded numbers and information consistency, largely depending on the reporting network and the surveillance effort. The extended Greek coastline and the numerous islands and islets can add further uncertainty to the recording of stranding incidents. However, the information used here provides a baseline for the regions and opens a path for future work to further investigate the drivers and relate with the seal population. Future work may involve assessing mortality grounds—the areas where death occurred based on stranding locations [11]. Such work could include running drifting models (e.g., the Leeway model) based on local circulation regimes. The output can assist in mapping stranding probabilities and visualizing patterns of high mortality areas associated with population areas [51,52]. Peltier et al. [11] even propose a population indicator, called stranding anomaly, based on the difference between expected and observed stranding events of the harbor porpoise in the North Sea. This could provide new insight into the importance of stranding records and contribute to the knowledge required for the institution of marine protected areas (MPAs) and supporting monitoring schemes.

## 5. Conclusions

Our results demonstrated that most of the Mediterranean monk seal strandings in the coastal areas of Greece fall into the category of “unknown” cause of death, being higher mainly during the winter period. Thus, further investigations that are going to shed light on the actual reasons behind strandings of this category are needed. Anthropogenic factors were shown to play an important role. Deliberate killings and fishing gear entanglements constitute the vast majority of “human-related” stranding incidents. In addition, the size of small-scale fishing grounds was found to be significantly associated with the possibility of a stranding event, providing an additional indication of the importance of the interaction with fishers. Certain areas in the Aegean and Ionian Seas were highlighted as stranding hotspot areas. Future conservation efforts should focus on these coastal areas, especially in the cases where human-related mortality seems to occur. 

For the first time, an analysis of the effect of oscillation indices on stranding events was carried out. A positive correlation was found regarding the effect of ENSO and NAO during the first half of the year and SST over the Greek Seas during the second half of the year. Additional research in this direction is encouraged. Finally, additional analysis was carried out to identify areas with high susceptibility to subadult strandings on a small-scale basis, focusing on the central part of the Ionian Sea. Similar analyses could be undertaken for wider areas, especially where reproduction is known to occur. 

## Figures and Tables

**Table 1:** Factors selected for inclusion in the MCDA analysis. For each factor, the correlation sign is shown. Factors with a positive sign (+) are positively correlated with the objective, meaning that the greater the attribute value, the higher the possibility of a stranding event. Factors with a negative sign (−) have the exact opposite effect. SSF = small-scale fisheries.

Factor	Correlation	Weight
Feeding grounds	Negative (−)	21.13%
(Distance from) breeding and disturbed caves	Negative (−)	21.13%
(Distance from) aquaculture	Negative (−)	4.94%
(Distance from) NATURA 2000 sites	Positive (+)	4.94%
SSF fishing grounds	Positive (+)	47.9%

**Table 2:** Categories of monk seal strandings that are considered “human-related” and the respective percentage from 2011 to 2021.

Cause of Death	Percentage (%)
Gunshot	20.9
Internal bleeding (Dynamite)	7.2
Net or fish hook entanglement	25.4
Serious one or multiple wounds (head, abdominal, pelvic, tail)	46.5

**Table 3:** MCDA analysis: The importance of the weights for each criterion was estimated using the pair-wise comparison matrix, produced by the AHP method. Here, 1 denotes that two factors are equally important, while 7 considers that one factor is much more important than the other. SSF = small-scale fisheries.

	Feeding Grounds	(Distance From) Breeding and Disturbed Caves	(Distance From) Aquaculture	(Distance From) NATURA 2000Sites	SSF Fishing Grounds	Weight
Feeding grounds	1	1	5	5	1/3	21.1%
(Distance from) breeding and disturbed caves	1	1	5	5	1/3	21.1%
(Distance from) aquaculture	1/5	1/5	1	1	1/7	4.9%
(Distance from) NATURA 2000 sites	1/5	1/5	1	1	1/7	4.9%
SSF fishing grounds	3	3	7	7	1	47.9%

## Data Availability

Monk seal strandings data were obtained from port authority reports and, according to the restrictions imposed by Greek legislation (Ministerial Decree 3376, Vol. B/19 May 2023) cannot be disclosed to the public.
